# Viral–Host Interactome Analysis Reveals Chicken STAU2 Interacts With Non-structural Protein 1 and Promotes the Replication of H5N1 Avian Influenza Virus

**DOI:** 10.3389/fimmu.2021.590679

**Published:** 2021-04-21

**Authors:** Qiao Wang, Qi Zhang, Maiqing Zheng, Jie Wen, Qinghe Li, Guiping Zhao

**Affiliations:** State Key Laboratory of Animal Nutrition, Institute of Animal Sciences, Chinese Academy of Agricultural Sciences, Beijing, China

**Keywords:** H5N1 AIV, AP-MS, Stau2, NS1, chicken

## Abstract

As a highly pathogenic influenza virus, H5N1 avian influenza virus (AIV) poses a great threat to poultry production and public health. H5N1 AIV has a small genome and, therefore, relies heavily on its host cellular machinery to replicate. To develop a comprehensive understanding of how H5N1 AIV rewires host cellular machinery during the course of infection, it is crucial to identify which host proteins and complexes come into physical contact with the viral proteins. Here, we utilized affinity purification mass spectrometry (AP-MS) to systematically determine the physical interactions of 11 H5N1 AIV proteins with host proteins in chicken DF1 cells. We identified with high confidence 1,043 H5N1 AIV–chicken interactions involving 621 individual chicken proteins and uncovered a number of host proteins and complexes that were targeted by the viral proteins. Specifically, we revealed that chicken Staufen double-stranded RNA-binding protein 2 interacts with AIV non-structural protein 1 (NS1) and promotes the replication of the virus by enhancing the nuclear export of *NS1* mRNA. This dataset facilitates a more comprehensive and detailed understanding of how the host machinery is manipulated during the course of H5N1 AIV infection.

## Introduction

Influenza A virus (IAV) is a segmented, single-stranded, negative-sense RNA virus that has adapted to infect multiple species. This virus causes annual epidemics and recurring pandemics, which have huge impacts on public health. IAV particles have two viral surface glycoproteins (hemagglutinin, HA; neuraminidase, NA) and one matrix-2 protein (M2). Inside the virion, all eight viral RNA (vRNA) segments bind three RNA polymerases (polymerase acid protein, PA; polymerase basic protein 1, PB1; and 2, PB2) and are encapsidated by the nucleoprotein (NP) to form the viral ribonucleoprotein (vRNP) complexes (1–5). The vRNPs are surrounded by a layer of the matrix protein, M1, which lines the envelope (6). Upon infection by influenza, the host cells detect the viral RNA through pathogen sensors, and the major gene products of the influenza virus mediate the viral life cycle and modulate cellular processes (7).

Viruses rely on host cellular functions to replicate, and thus, they hijack the host cell machinery and rewire it for their own needs. Several proteomic studies have used affinity purification mass spectrometry approaches to identify a series of cellular factors that interact with IAV proteins (8–10). However, knowledge of common and strain-specific interactions remains incomplete, and how these interactions control host defense and viral infection remains to be fully elucidated. A comprehensive understanding of host–virus interactions would greatly improve our understanding of the viral life cycle and host resistance mechanisms. Here, we applied the AP-MS technology to uncover a wide array of host proteins, complexes, and pathways that are hijacked by H5N1 avian influenza virus (AIV) during the course of infection. We constructed an H5N1 AIV–chicken protein interaction map, and in addition to replicating previously identified host factors, we uncovered several novel interactions. Among the novel factors, chicken Staufen double-stranded RNA-binding protein 2 (STAU2) was found to be a crucial component when the viral mRNA is transported during the replication stage of the viral life cycle. Importantly, STAU2 interacts with the influenza NS1 protein and promotes the replication of H5N1 AIV by promoting the transport of *NS1* mRNA from the nucleus to the cytoplasm.

## Materials and Methods

### Cells and Virus

Chicken embryonic fibroblast (DF1) cells, Madin–Darby canine kidney (MDCK) cells, and human embryonic kidney cells (293T) were cultured in Dulbecco's modified Eagle's medium (DMEM) supplemented with 10% fetal bovine serum (Gibco), 100 μg/ml streptomycin, and 100 U/ml penicillin at 37°C under a humidified atmosphere of 5% CO_2_. The highly pathogenic H5N1 strain A/mallard/Huadong/S/2005 (SY) (11) was propagated in 10-day-old specific pathogen-free embryonic chicken eggs. The H5N1 influenza virus strain 178 (GenBank Accession No. AY737296-737300) was isolated from a chicken in Guangdong, China, by the MOA Key Laboratory for Animal Vaccine Development, P.R. China. The experiments that involved live viruses were performed in a biosafety cabinet with HEPA filters in a biosafety level 3 laboratory in Yangzhou University or South China Agricultural University.

### Plasmid Construction

The H5N1 AIV genes were amplified by high-fidelity DNA polymerase (TransGen), and cDNA derived from the H5N1 virus (A/Chicken/ShanXi/2/2006) was used as the template. To construct the FLAG-tagged C-terminus fusion proteins, a 3 × FLAG tag was inserted into the C-terminus of the pcDNA 3.1 vector, and the *PA, PB1, PB2, NP, HA, NA, M1*, and *NS1* genes were cloned upstream of the tag using the Seamless Assembly Cloning Kit (CloneSmarter). For the GFP-tagged proteins, a GFP tag was inserted into the C-terminus of the pcDNA 3.1 vector, and the *NS2, M2*, and *PB1-F2* genes were cloned upstream of the GFP tag using the same method. All the expression vectors were validated by sequencing.

### Antibodies

The following antibodies were used in this study: anti-FLAG [Abmart, # M20008L, WB (1:2,000)], anti-Myc [Abmart, # M20002L, WB (1:2,000)], and anti-Lamin B1 [Abcam, # ab16048, WB (1:2,000)].

### Protein Co-immunoprecipitation and Western Blotting

The transiently transfected cells were washed twice with phosphate-buffered saline (PBS) and were then lysed in radioimmunoprecipitation assay (RIPA) buffer (50 mM Tris–HCl, pH 7.4, 150 mM NaCl, 0.25% deoxycholic acid, 1% NP-40, 1 mM EDTA, and 0.5% SDS supplemented with protease inhibitor, Roche). The whole-cell lysate was firstly precleared with a protein A/G slurry (Millipore) and was then incubated with 40 μl of the anti-Flag affinity gel (Sigma-Aldrich) at 4°C for 2 h or with 10 μl of the GFP antibody at 4°C overnight, and then, it was incubated with 40 μl of the protein A/G slurry (Millipore) at 4°C for 2 h. The immunoprecipitated samples were washed four times with RIPA buffer and twice with 54K buffer (50 mM Tris–HCl, pH 7.4, 150 mM NaCl, and 0.25% Triton X-100 supplemented with protease inhibitor). The FLAG tag-associated proteins were eluted with 250 ng/μl of the Flag peptide (Sigma) by rocking the samples on a tilted rotator at 4°C for 2 h. The GFP tag-associated proteins were eluted with ammonium hydroxide at 4°C for 2 h, and the supernatant was collected with a vacuum centrifugal concentrator.

For Western blotting, SDS electrophoresis was performed, and the separated proteins were transferred to polyvinylidene fluoride (PVDF) membranes. The membranes were blocked and then incubated with the corresponding antibodies. The proteins were visualized using the Immobilon Western Chemiluminescent HRP Substrate (Millipore).

### Mass Spectrometry

Strict experimental controls were used for the MS analysis. The immunoprecipitated samples from the empty FLAG-transfected cells (empty FLAG control)/empty GFP-transfected cells (empty GFP control) and the protein complexes that were pulled down by the normal IgG (IgG control) were also subjected to mass spectrometry for identification. All the proteins identified in these two sets of controls were excluded from consideration as H5N1 AIV interacting proteins. The authentic FLAG-precipitated proteins and the GFP-precipitated proteins associated with H5N1 AIV were examined in triplicate. Proteins that were enriched by co-immunoprecipitation were separated by SDS-PAGE, and the entire lane was cut and sent for tryptic digestion.

Proteins pulled down by the anti-FLAG beads or the anti-GFP antibody were digested with trypsin for 20 h at room temperature. The peptides were extracted twice with 50% aqueous acetonitrile containing 0.1% formic acid, dried in a SpeedVac, and then desalted using Sep-Pak C18 cartridges. Tandem mass tag (TMT) reagents (Thermo Fisher) were used to label the purified peptides according to the manufacturer's instructions. Briefly, the TMT labeling reagents, in anhydrous acetonitrile, were carefully added to the desalted peptides and incubated for 1 h at room temperature, after which the reactions were stopped by hydroxylamine, and the TMT-labeled peptides separated by reverse phase (RP) chromatography. The first dimension RP separation by micro-liquid chromatography (LC) was performed on an Ultimate 3000 System (Thermo Fisher) using an Xbridge C18 RP column (5 μm, 150 Å, 250 mm × 4.6 mm i.d., Waters). The samples were reconstituted in 5–10 ml of mobile phase A (2% acetonitrile, 0.1% formic acid, pH adjusted to 10.0 with NH_4_OH). The peptides were monitored at 214 nm, and 1-min fractions were collected, dried, and reconstituted in 20 μl of 0.1% (v/v) formic acid in water for the nano-LC-MS/MS analyses, in which the fractions were further separated on a C18 column (75 μm inner diameter, 150 mm length) with a flow rate of 250 nl/min. A gradient was formed, and the peptides were eluted with increasing concentrations of solvent B (98% acetonitrile, 0.1% formic acid, pH to 10.0, as above). This second-dimension separation was performed on an Orbitrap Q Exactive mass spectrometer that was operated in data-dependent acquisition mode using Xcalibur 3.0 software. The scan range was from *m*/*z* 300 to 1,800, with a resolution of 70,000 at *m*/*z* 400. A full scan followed by 20 data-dependent MS/MS scans was acquired with collision-induced dissociation having a normalized collision energy of 35%. The MS/MS spectra obtained from each LC-MS/MS run were searched against a protein database using the Proteome Discoverer searching algorithm. The precursor ion mass tolerance was set at 20 ppm, and the fragment ion mass tolerance was 20 mmu. One missed cleavage by trypsin was allowed. Oxidation (Met) was chosen as the variable modification, and carbamidomethyl (Cys) and TMT6plex were chosen as the fixed modifications.

#### Significance Analysis of Interactome

The MS data were analyzed using the Significance Analysis of Interactome (SAINT) tool, and only the results with a SAINT score >0.9 were considered for the subsequent analysis. To define the high-probability interaction sets, we selected the interactions at a probability threshold of 0.9, which was approximately equivalent to an estimated false discovery rate (FDR) of 2% (12).

### Gene Ontology Term Enrichment and Pathway Enrichment Analysis

Gene Ontology (GO) enrichment and pathway enrichment were conducted using the Database for Annotation, Visualization and Integrated Discovery (DAVID) (13).

### Domain Enrichment Analysis

The protein domain enrichment analysis was conducted for the H5N1 AIV-associated host proteins using FunRich (http://funrich.org/index.html).

### Immunofluorescence Staining and Confocal Microscopy Analysis

Chicken DF1 cells transiently transfected with FLAG-NS1 and Myc-STAU2 plasmids were cultured for 24 h and were then fixed in 4% paraformaldehyde for 30 min at room temperature and were permeabilized with 0.1% Triton X-100 (Sigma, # T8787) for 15 min. FLAG-NS1 cells were incubated with an anti-FLAG antibody (Abmart, # M20008L) and Myc-STAU2 cells with an anti-Myc antibody (Abmart, # M20002L), both at a 1:1,000 dilution for 2 h. After three washes in PBS, the cells were subsequently incubated with a FITC-conjugated secondary antibody (Abcam, # ab6785) for FLAG and with a Cy3.5-conjugated secondary antibody (Abcam, # ab6954) for Myc, both at a 1:1,000 dilution for 1 h. The nuclei were stained with 6-diamidino-2-phenylindole (DAPI) (Sigma, # D9542, 1:500). Finally, the cells were visualized with a confocal microscope (Nikon A1 R MP).

### RNA Interference

All the small interfering RNAs (siRNAs) used in this study were designed and synthesized by Guangzhou Ruibo (Guangzhou, China). Chicken DF1 cells, at 90% confluence in six-well plates, were transfected with 100 nM of an effective siRNA specific for the chicken *STAU2* gene (Gene ID: 420184: siSTAU2, sense 5′-CCTACAAGCTCTCCAGAAT-3′; siNC, sense 5′-GUGAACGAACUCCUUAAUUTT-3′). Human 293T cells, at 65% confluence in six-well plates, were transfected with 100 nM of an effective siRNA specific for the human *STAU2* gene (Gene ID: 27067: siSTAU2, sense 5′-CCAAGGGAUGAACCCUAUUTT-3′). All the siRNAs were transfected into the cells using Lipofectamine 3000 (Life Technologies).

### Cell Culture and Viral Transfections

The highly pathogenic H5N1 strain A/mallard/Huadong/S/2005 (YS), showing a high virulence in mice, was isolated from a mallard. Chicken DF1 embryonic fibroblast cells, 293T cells, and MDCK cells were cultured in DMEM supplemented with 10% fetal bovine serum (Gibco), 100 μg/ml streptomycin, and 100 units/ml penicillin at 37°C under a humidified atmosphere of 5% CO_2_. The transfection with siRNA or plasmid was performed with Lipofectamine 3000 (Life Technologies). The cells were harvested for protein extraction 48 h after the transfection.

### Virus Titration

The 50% tissue culture infectious dose assay (TCID_50_) was used to evaluate progeny virus production. The siRNA-STAU2- or siRNA-NC-transfected DF1 cells grown in six-well plates were infected with H5N1 AIV, A/mallard/Huadong/S/2005 (SY), at a multiplicity of infection (MOI) of 0.1. After a 1-h incubation, the supernatants were discarded, and the cells were washed twice with PBS. Subsequently, 2 ml of DMEM without fetal bovine serum was added. The supernatants were collected at 12 and 18 h postinfection (h.p.i.), and the virus titers were determined in the MDCK cells. The virus titer was calculated as the TCID_50_ per 0.1 ml using the Reed and Muench method. The data are shown as the means ± standard deviations (SD) from three independent experiments. An independent-sample *t*-test was used to analyze the TCID_50_ results. For all the tests, *P* ≤ 0.05 was considered significant.

### Extraction of Nuclear and Cytoplasmic RNA

The DF1 cells were transfected with an effective siRNA specific for *STAU2* for 12 h and then were transfected with the NS1-FLAG plasmid for 24 h. The DF1 cells and 293T cells were infected with the H5N1 avian influenza virus for 10 h. The fresh cells were collected and were then separated into the nuclear and cytoplasmic fractions to extract the nuclear RNA and cytoplasmic RNA, respectively, using the PARIS™ Kit (Life Technologies, USA).

### Real-Time PCR

Total cellular RNA was extracted by TRIzol (Invitrogen) according to the manufacturer's instructions. Next, cDNA was generated from 1 μg of RNA using the PrimeScript RT reagent kit with gDNA Eraser (TaKaRa). The target mRNAs were quantified by real-time PCR using SYBR Green Master Mix. The data were normalized to the expression of the housekeeping genes *GAPDH* and β*-actin. NS1* expression in the nucleus was normalized to the expression of *U6*, which is only expressed in the nucleus. The sequences of the PCR primers used to amplify target genes are listed as follows: *STAU2-*chicken: sense 5′-AGTGCCTAAAATTTTCTATGT-3′, antisense 5′-GCTTCCCCATTCTGAGGTAAT-3′; *STAU2-*human: sense 5′-AAGCACTGCAGAATGAA-3′, antisense 5′-GCAATTTCAAACACTAA-3′; *NS1*: sense 5′-TGCGGGAAAGCAGATAGT-3′, antisense 5′-TGGGCATGAGCATGAACC-3′; *GAPDH-*chicken: sense 5′-CCCCCATGTTTGTGATGGGT-3′, antisense 5′-TGATGGCATGGACAGTGGTC-3′; *GAPDH-*human: sense 5′-AACCATGAGAAGTATGAC-3′, antisense 5′-GATGGCATGGACTGTGGT-3′; β*-actin-*chicken: sense 5′-GAGAAATTGTGCGTGACATCA-3′, antisense 5′-CCTGAACCTCTCATTGCCA-3′; β*-actin-*human: sense 5′-CTCCATCCTGGCCTCGCTGT-3′, antisense 5′-GCTGTCACCTTCACCGTTCC-3′; *U6-*chicken: sense 5′-CTCGCTTCGGCAGCACATAT-3′, antisense 5′-TGGAACGCTTCACGAATTTG-3′; and *U6-*human: sense 5′-TTCGGCAGCACATATA-3′, antisense 5′-ATATGGAACGCTTCAC-3′.

## Results

### Identification of Host Proteins Associated With 11 Viral H5N1 AIV Proteins

**Figure 1:**
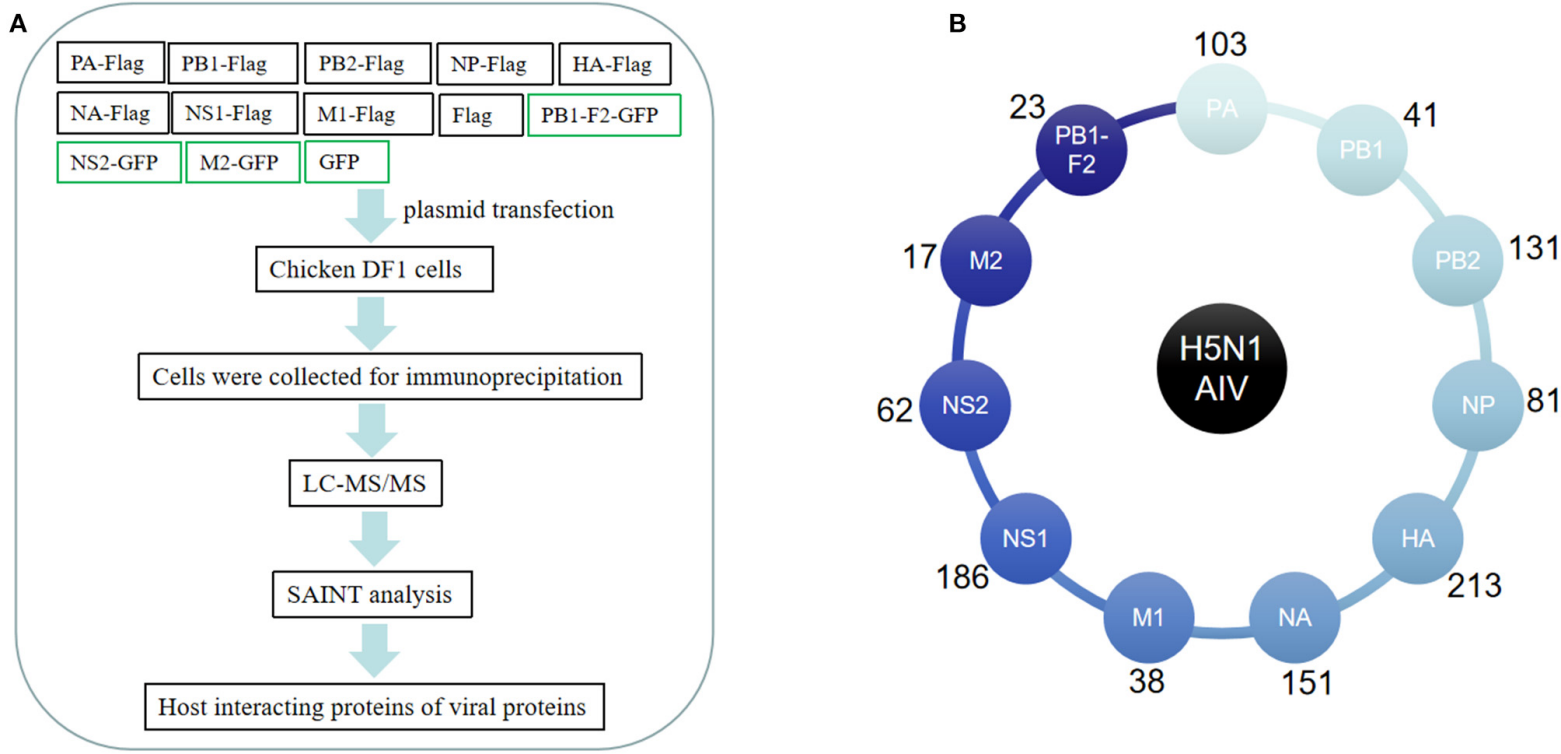
Affinity purification of H5N1 avian influenza virus (AIV) proteins. **(A)** Flowchart of the proteomic affinity purification mass spectrometry (AP-MS) method used to define the H5N1 AIV–host interactome. **(B)** The number of host proteins identified by AP-MS that interact with individual H5N1 avian influenza virus proteins.

We aimed to systematically and quantitatively identify host proteins associated with H5N1 AIV proteins using AP-MS. To this end, eight FLAG-tagged viral proteins (i.e., PA, PB1, PB2, NP, HA, NA, M1, and NS1) and three GFP-tagged viral proteins (i.e., PB1-F2, M2, and NS2) of an avian influenza virus (A/Chicken/ShanXi/2/2006, H5N1 subtype) were individually expressed in chicken DF1 cells. Affinity purification was carried out using FLAG tag or GFP tag antibodies to immunoprecipitate the host proteins associated with the tagged viral proteins. The Western blot results showed that all the viral genes were expressed in the chicken cells (Supplementary Figure 1). The immunoprecipitated samples from the plasmid-transfected cells were separated by SDS-PAGE for MS analysis (Figure 1A). In order to generate AP-MS data with a high degree of confidence, all the samples (including those from the empty FLAG/GFP-transfected cells and the IgG control samples) were subjected to the SAINT analysis (12), and only the results with a SAINT score >0.9 were considered for the subsequent analysis. The MS analysis of the coprecipitated proteins identified 621 host proteins in total, and among these, 103, 41, 23, 131, 81, 213, 151, 38, 17, 186, and 62 coprecipitated with the viral PA (14), PB1, PB1-F2, PB2, NP, HA, NA, M1, M2, NS1, and NS2 proteins, respectively (Figure 1B, Supplementary Table 1).

### H5N1 AIV–Chicken Interactome

**Figure 2:**
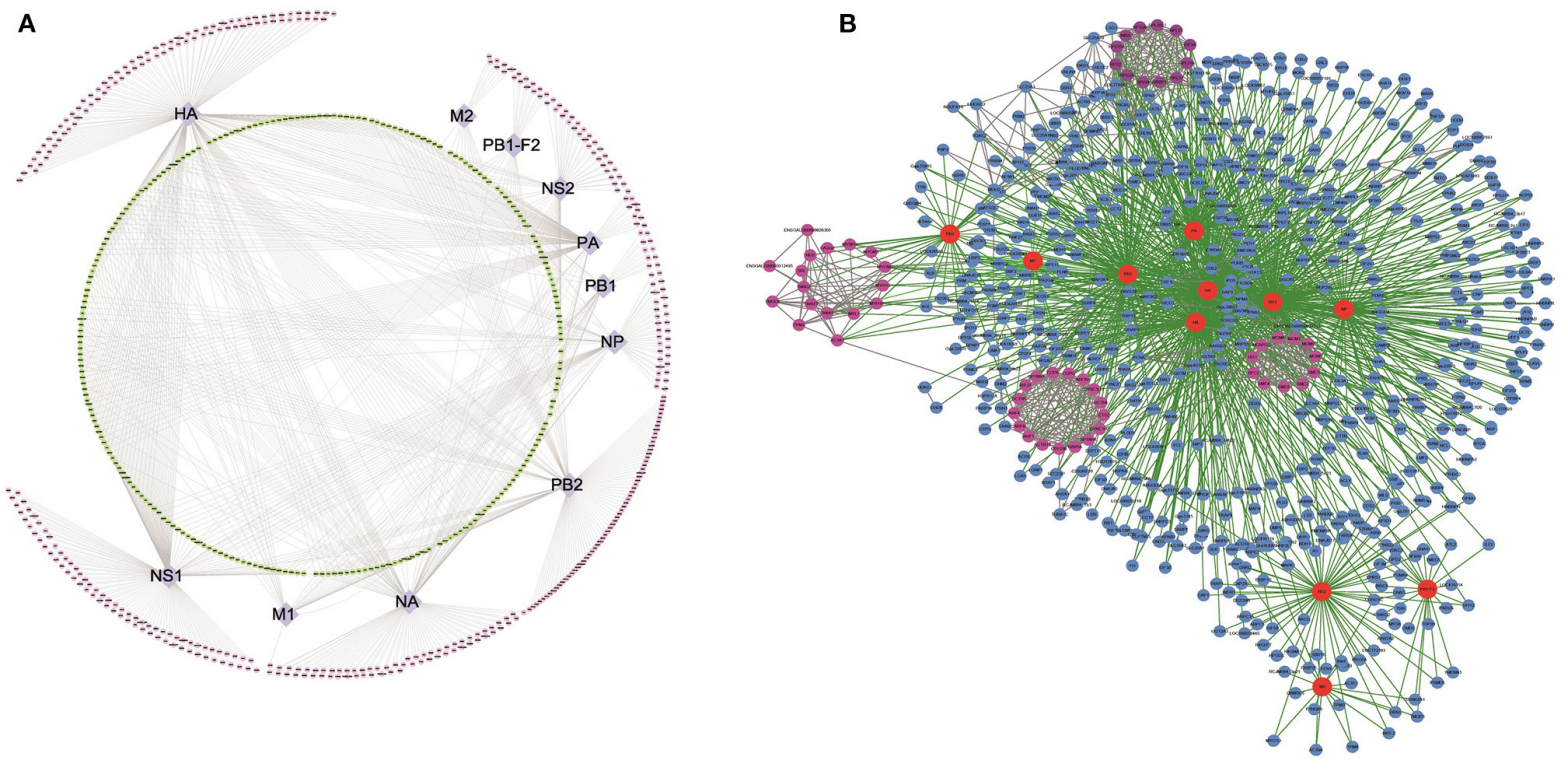
Global landscape of the H5N1 AIV–chicken protein complexes. In total, 1,043 H5N1 AIV–chicken interactions are represented, linking 11 H5N1 AIV proteins and 621 chicken factors. **(A)** Types of host interacting proteins. Chicken proteins that interact with H5N1 AIV proteins (purple nodes in the middle ring) are denoted with two colors: the pink nodes in the outer ring represent the host proteins that interact with only one specific virus protein, while the green nodes in the inner ring represent the host proteins that interact with a variety of viral proteins. **(B)** Network representation of the H5N1 AIV–human PPIs. The host–pathogen interaction map for H5N1 AIV contained 1,475 interactions between 11 influenza virus proteins (red nodes) and 621 cellular proteins (blue and pink nodes). Some of the host proteins interact with the virus in the form of protein complexes (pink nodes).

We next plotted a network representation of the 621 H5N1 AIV–chicken protein interactions identified in this study (Figure 2A). This network contained nodes corresponding to 11 H5N1 AIV proteins (purple) and the 621 host proteins derived from the chicken DF1 cells (pink and green). The outermost pink nodes and the innermost green nodes represent the host proteins that interact with only one viral protein or with multiple viral proteins, respectively. The network structure suggests that a large proportion of the interacting host proteins are not only factors for this particular influenza virus but may have more general biological functions and interact with multiple viral proteins.

Next, we analyzed all of the H5N1 AIV–host protein interactions using Search Tool for the Retrieval of Interacting Genes/Proteins (STRING) (15) and plotted a network representation (Figure 2B, Supplementary Table 2) incorporating both the MS and STRING analyses (Supplementary Table 3) using Cytoscape. In this network, the red nodes represent the viral proteins, the remaining are the host interaction proteins, and the pink nodes form the subnetworks in the protein–protein interaction (PPI) map. The green lines represent the interactions among the virus and host proteins, while the gray lines represent interactions among the host proteins. In addition to interactions with the viral proteins, it was evident that many of the host interacting proteins also interacted with the other host proteins, and some even associated with the viral proteins in the form of protein complexes (Figure 2B).

### GO, Pathway, and Domain Enrichment Analysis of the Host Interacting Partners of H5N1 AIV

We performed a GO analysis and pathway enrichment on the host interactors of the H5N1 AIV proteins using the DAVID analysis tool. A number of crucial GO terms and pathways were identified as enriched in the AIV–chicken interacting proteins (Tables 1, 2), indicating that these signaling pathways and associated genes play important roles in the life cycle of the influenza virus.

**Table 1:** Gene Ontology terms enriched among host proteins interacting with H5N1 AIV.

**GO term**	**Count**	**Involved genes/total genes (%)**	***P*-value**
**Molecular function**			
Poly(A) RNA binding	116	22.4	1.30E-37
ATP binding	106	20.5	2.50E-15
RNA binding	37	7.1	1.10E-11
Nucleotide binding	35	6.8	4.40E-10
ATP-dependent RNA helicase activity	13	2.5	4.80E-07
Actin filament binding	13	2.5	1.10E-06
Structural constituent of ribosome	21	4.1	2.30E-05
mRNA binding	14	2.7	2.70E-05
Translation initiation factor activity	10	1.9	5.20E-05
Double-stranded RNA binding	9	1.7	3.30E-04
**Cellular component**			
Extracellular exosome	149	28.8	2.90E-22
Membrane	86	16.6	1.40E-19
Myelin sheath	29	5.6	2.30E-13
Nucleoplasm	89	17.2	7.40E-11
Focal adhesion	39	7.5	1.10E-10
Catalytic step 2 spliceosome	16	3.1	5.60E-08
Mitochondrial inner membrane	23	4.4	4.10E-07
U1 snRNP	7	1.4	3.90E-06
Nucleolus	40	7.7	7.50E-06
U5 snRNP	7	1.4	1.30E-05
U2 snRNP	7	1.4	1.30E-05
**Biological process**			
RNA secondary structure unwinding	10	1.9	3.90E-06
Translation	20	3.9	1.50E-05
Protein folding	15	2.9	2.80E-05
IRES-dependent viral translational initiation	5	1	3.00E-05
mRNA splicing, via spliceosome	11	2.1	2.20E-04
Negative regulation of translation	8	1.5	3.60E-04
Osteoblast differentiation	11	2.1	4.00E-04
Viral translational termination-reinitiation	4	0.8	4.80E-04
mRNA processing	10	1.9	7.40E-04
Regulation of translational initiation	6	1.2	8.50E-04
Protein import into nucleus	8	1.5	1.00E-03

**Table 2:** Pathway enrichments of host proteins interacting with H5N1 AIV.

**Term**	**Count**	**Involved genes/total genes (%)**	***P*-value**
Spliceosome	28	5.4	8.40E-11
Proteasome	11	2.1	4.40E-05
RNA transport	22	4.2	4.50E-05
Citrate cycle (TCA cycle)	9	1.7	2.10E-04
Ribosome	19	3.7	3.00E-04
DNA replication	9	1.7	3.60E-04
Carbon metabolism	16	3.1	7.40E-04
Protein processing in endoplasmic reticulum	20	3.9	1.50E-03
Pyruvate metabolism	8	1.5	4.20E-03
*Salmonella* infection	11	2.1	7.00E-03
Biosynthesis of antibiotics	21	4.1	8.50E-03
Aminoacyl-tRNA biosynthesis	8	1.5	1.40E-02
Ribosome biogenesis in eukaryotes	10	1.9	2.00E-02
Mismatch repair	5	1	3.20E-02
Pentose phosphate pathway	5	1	4.40E-02

Protein domains provide insight into the preferential structure of an interaction partner. To survey the binding preferences of the H5N1 AIV viral proteins, we carried out a protein domain enrichment analysis on the H5N1 AIV-associated host protein dataset using FunRich (http://funrich.org/index.html) (Table 3). Multiple protein domains were identified as significantly enriched among the H5N1 AIV-associated host proteins, including the RRM, AAA, SM00913, DEXDc, HELICc, and MCM domains (Table 3). MCM is required for the initiation of eukaryotic DNA replication (16, 17), while HELICc is the C-terminal domain found in proteins belonging to the helicase superfamilies 1 and 2. These families encompass a large number of DNA and RNA helicases from archaea, eubacteria, eukaryotes, and viruses (18, 19). Proteins with the Sm domain are involved in processing pre-mRNAs to mature mRNAs that are part of specific small nuclear ribonucleoproteins (snRNPs) and are a major component of the eukaryotic spliceosome (20). Altogether, the proteins containing the abovementioned domains may be more inclined to interact with viral proteins and, thus, factor into the replication of the virus.

**Table 3:** Enriched protein domains among host proteins interacting with H5N1 AIV.

**Term**	**Count**	**Involved genes/total genes (%)**	***P*-value**
RRM	33	6.4	1.40E-14
AAA	22	4.2	1.00E-08
SM00913	6	1.2	1.30E-05
DEXDc	14	2.7	4.70E-05
HELICc	14	2.7	4.70E-05
MCM	5	1	1.70E-04
RRM_1	6	1.2	1.70E-03
SM00968	4	0.8	2.00E-03
CH	8	1.5	2.40E-03
PINT	5	1	3.70E-03
PHB	4	0.8	7.70E-03
SM00991	3	0.6	1.30E-02
MYSc	6	1.2	1.40E-02
SM00847	4	0.8	2.30E-02
Sm	4	0.8	2.80E-02
KH	5	1	3.50E-02

### Comparative Analysis of the Host Proteins That Interact With the H5N1 AIV and H1N1 Influenza Viruses

**Figure 3:**
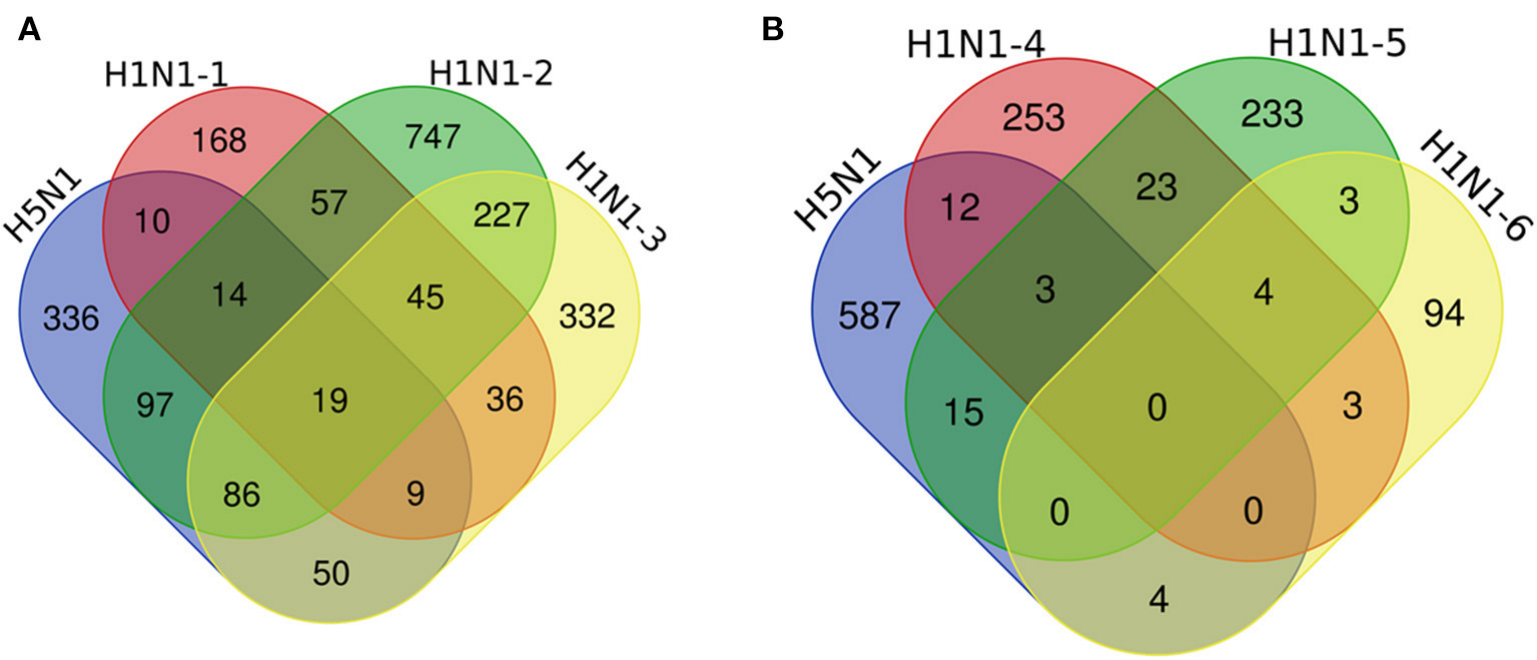
Comparative analysis of the host proteins that interact with H5N1 AIV and H1N1 influenza viruses. Venn diagrams illustrate the overlap of the host interacting proteins between our data and other IAV-related datasets, including previously published H1N1 IAV–human PPIs **(A)** and host proteins implicated in H1N1 IAV function by an RNA interference (RNAi) screen **(B)**. H5N1 indicates our data, A/mallard/Huadong/S/2005 (SY), chicken DF1 cells; H1N1-1, influenza A/Puerto Rico/8/34, human HEK293 cells; H1N1-2, A/WSN/33, human HEK293 cells; H1N1-3, A/Puerto Rico/8/1934, human A549 cells; H1N1-4, WSN-Ren, human A549 cells; and H1N1-5 and H1N1-6, A/WSN/33, human A549 cells.

We compared our data to other IAV-related datasets, including previously published H1N1 IAV–human PPIs (Figure 3A, Supplementary Table 4) (8–10) and host proteins implicated in H1N1 IAV replication based on RNA interference (RNAi) screening (Figure 3B) (21–23). The results showed that 285 of the 621 host interacting proteins identified in this study were also host interacting factors for the H1N1 influenza virus (Figure 3A, Supplementary Table 4). We then analyzed the overlapping protein interactions using STRING and plotted a network representation (Supplementary Figure 2). It was obvious that there were close interactions between the H1N1 and H5N1 overlapping interacting proteins, which indicated that these host proteins tended to function in complexes. We further performed the GO analysis (Supplementary Table 5) and pathway enrichment analysis (Supplementary Table 6) on the overlapping interacting proteins using the DAVID analysis tool. The results revealed that these proteins were enriched for molecular functions, such as RNA binding, mRNA binding, and translation initiation factor activity, for biological processes, such as translation, DNA repair, protein binding, and signaling pathways, and for components of the spliceosome, ribosome, RNA transport, and the proteasome, thereby suggesting potential roles for these overlapping proteins in infection by H1N1 and H5N1. Furthermore, 34 of the host interacting proteins identified in this work significantly affected the replication of the H1N1 influenza virus (Figure 3B), including ARCN1, COPG, APOA1, CSE1L, MIB1, and PSMD2.

### STAU2 Interacts and Colocalizes With AIV NS1

**Figure 4:**
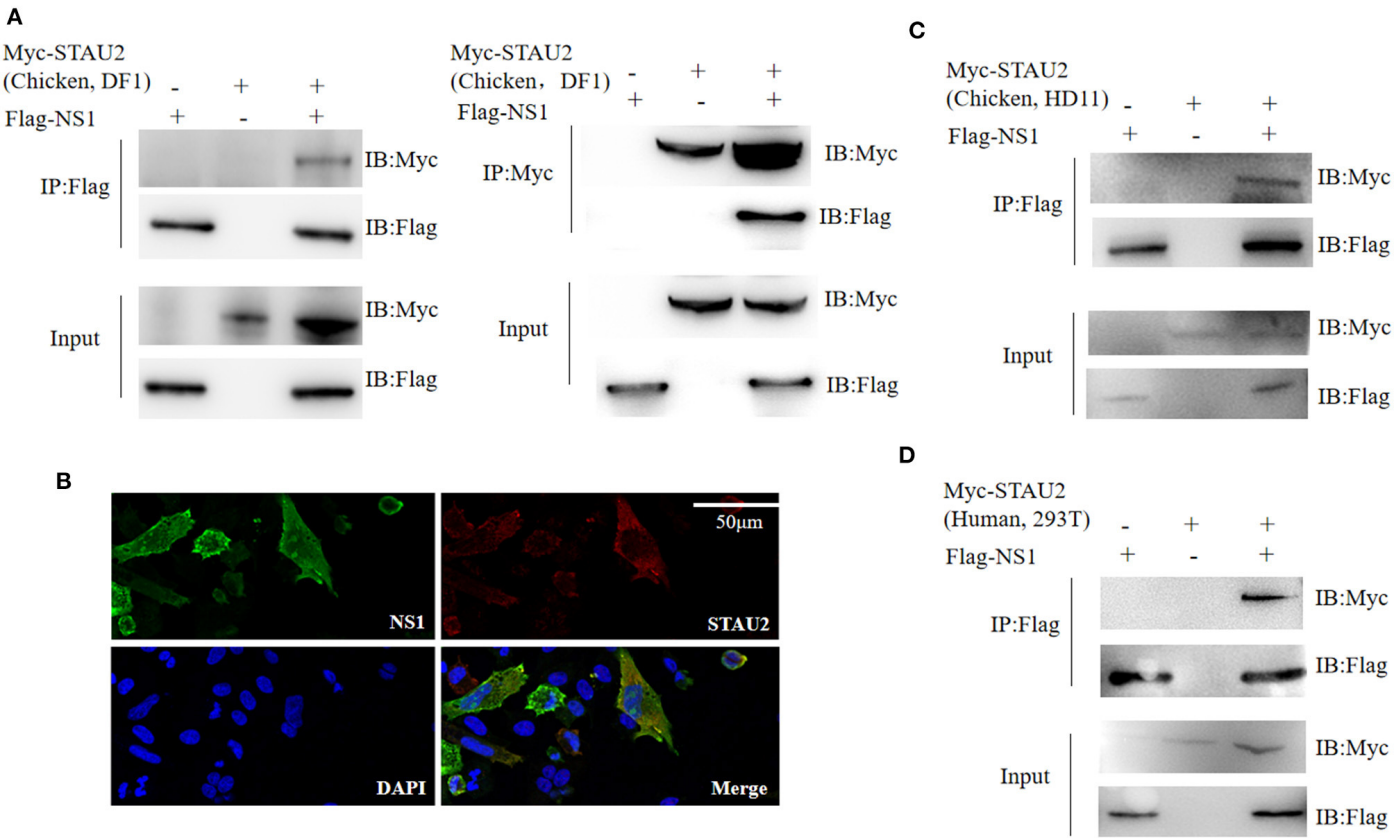
Interaction of Staufen double-stranded RNA-binding protein 2 (STAU2) with non-structural protein 1 (NS1). **(A)** Chicken DF1 cells were transfected with the indicated plasmids. An immunoprecipitation was carried out to detect the interaction between STAU2 and NS1 using anti-FLAG or anti-Myc antibodies, followed by an immunoblot analysis with the indicated antibodies. **(B)** An immunofluorescence analysis of STAU2 and NS1. Chicken DF1 cells transfected with FLAG-tagged NS1 and Myc-tagged STAU2 were fixed and incubated with anti-FLAG and anti-Myc antibodies, followed by an incubation with the secondary antibody. The nuclei were stained with DAPI. The colocalization of NS1 and STAU2 was detected by confocal microscopy. **(C,D)** Confirmation of the STAU2–NS1 interaction in additional cell types. Chicken HD11 cells or human 293T cells were transfected with the indicated plasmids. An immunoprecipitation using anti-FLAG followed by an immunoblot analysis with the indicated antibodies was carried out to detect the interaction between STAU2 and NS1.

In the nucleus of a virus-infected cell, the polymerase transcribes the viral genome into mRNA, which is then transported back to the cytoplasm and translated into viral proteins. Thus, the transport of viral mRNA from the nucleus to the cytoplasm is critical for the life cycle of the virus. Based on our AP-MS data, STAU2 was found to be a high-confidence interactor with the viral protein NS1 (Supplementary Table 1). Therefore, we investigated the association between NS1 and STAU2 by constructing expression vectors containing the encoding genes and transfecting them into chicken DF1 cells. As expected, exogenously introduced STAU2 interacted with NS1 *in vitro* (Figure 4A). Furthermore, an immunofluorescence analysis consistently demonstrated the colocalization of NS1 and STAU2 in chicken cells (Figure 4B). Taken together, these data suggested that STAU2 interacted and colocalized with NS1 in chicken DF1 cells. To further verify whether STAU2 interacted with H5N1 in a host cell-dependent manner, we verified the interaction between NS1 and STAU2 in chicken HD11 macrophages (Figure 4C) and human 293T cells (Figure 4D), and the immunoprecipitation assays confirmed that NS1 was bound with STAU2 in both cell types.

### STAU2 Promotes the Nuclear Transport of NS1 mRNA and Thereby Promotes the Replication of H5N1 AIV

**Figure 5:**
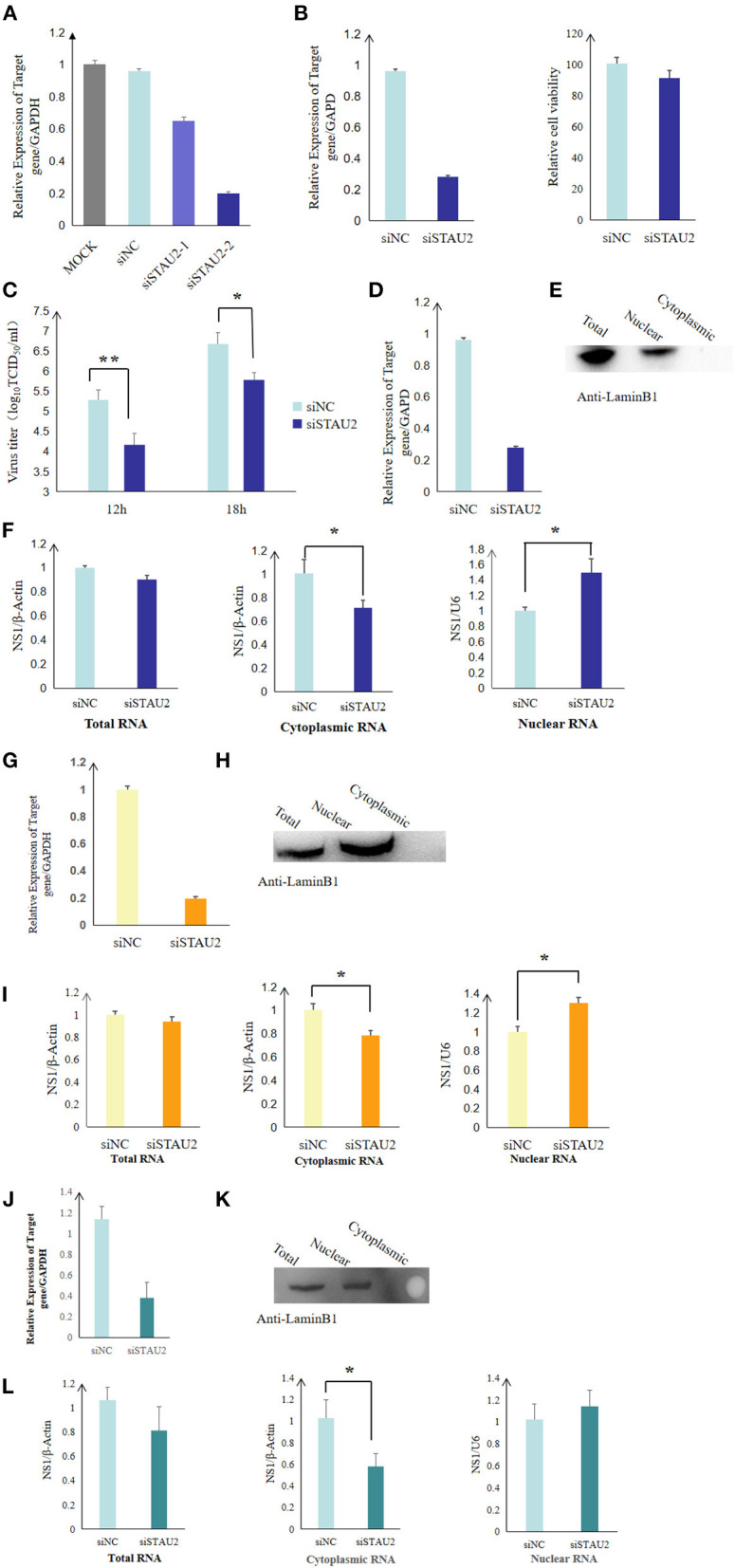
STAU2 promotes the nuclear transport of *NS1* mRNA and thereby promotes the replication of H5N1 AIV. The expression of *STAU2* was dramatically reduced in the specific siRNA-treated DF1 cells **(A)**, and its downregulation had no major effect on cell viability **(B)**. **(C)** Progeny virus titers after transfecting DF1 cells with siSTAU2 and infecting them with H5N1 AIV for 12 or 18 h. Chicken DF1 cells were treated with siSTAU2 or siNC for 12 h, which dramatically reduced the expression of *STAU2* **(D,G)**. The cells were then transfected with Flag-tagged NS1 for 24 h or infected with H5N1 AIV for 12 h. The DF1-treated cells were separated into their nuclear and cytoplasmic fractions, and this was confirmed by Western blotting with mouse pAb against LaminB1 **(E,H)**, and the distribution of *NS1* mRNA between the nuclear and cytoplasmic fractions was detected by RT-PCR **(F,I)**. The effect of STAU2 on *NS1* mRNA transportation was verified in 293T cells **(J–L)**.

To study the role of the STAU2–NS1 interaction in the virus life cycle, we analyzed the effect of *STAU2* downregulation on H5N1 AIV infection by utilizing siRNA-mediated silencing. Real-time PCR confirmed that the expression of *STAU2* was dramatically reduced in specific siRNA-treated DF1 cells but not in the cells treated with the scrambled siRNA (Figure 5A). *STAU2* downregulation had no major effect on cell viability as measured by a luminescent cell viability assay (Figure 5B). Subsequently, chicken DF1 cells treated with an siRNA targeting *STAU2* or with the scrambled siRNA were infected with H5N1 AIV. The culture supernatants were collected at 12 and 18 h.p.i. and were titrated in MDCK cells. As shown in Figure 5C, the knockdown of *STAU2* by a specific siRNA decreased the virus titer relative to the scrambled siRNA-treated DF1 cells. These data demonstrated that STAU2 positively regulated H5N1 AIV replication.

Viral mRNA localization is an essential mechanism for AIV replication, and Staufen (Stau) proteins are core factors of mRNA localization particles (24). To investigate whether STAU2 promotes the export of H5N1 IAV *NS1* mRNA from the nucleus to the cytoplasm, we first measured the distribution of *NS1* mRNA in the context of *STAU2* knockdown by RT-PCR. Chicken DF1 cells were treated with an siRNA targeting *STAU2* or with the scrambled siRNA for 12 h (Figure 5D), and then, the cells were transfected with Flag-tagged *NS1* for 24 h. The DF1-treated cells were separated into their nuclear and cytoplasmic fractions (Figure 5E), and RNA was extracted from each part to detect the distribution of the *NS1* mRNA (Figure 5F). There was no significant difference in the total amount of *NS1* mRNA extracted from the DF1 cells treated with the specific or scrambled siRNA. However, the downregulation of *STAU2* significantly reduced the level of cytoplasmic *NS1* mRNA while increasing nuclear *NS1* mRNA, indicating that STAU2 promoted the nuclear export of *NS1* mRNA (Figure 5F). To confirm the effect of STAU2 on endogenous viral *NS1* mRNA, we treated chicken DF1 cells with an siRNA targeting *STAU2* or with the scrambled siRNA for 12 h and then infected the cells with H5N1 AIV. RNA was again extracted from the nuclear and cytoplasmic fractions of the infected cells, and the distribution of the *NS1* mRNA was determined. The results were the same as with exogenous *NS1* (Figures 5G–I), further demonstrating that STAU2 promoted the export of *NS1* mRNA from the nucleus to the cytoplasm and thereby promoted the replication of H5N1 AIV.

We also verified the effect of STAU2 on *NS1* transportation in human cells. We knocked down human *STAU2* with an siRNA in H5N1-infected 293T cells and examined the abundance of viral mRNA in the cytoplasm and nucleus by RT-PCR. Consistent with our finding in chicken cells, the knockdown of *STAU2* in human cells efficiently blocked the nuclear export of *NS1* mRNA (Figures 5J–L) but did not affect the nuclear export of other viral mRNAs (Supplementary Figure 3).

## Discussion

Many studies have applied a variety of strategies to search for the host factors involved in viral replication (10, 22, 23, 25). Here, we present the interactome of the H5N1 avian influenza virus and chicken embryonic cells using an affinity tagging/purification approach. In summary, we identified 621 chicken proteins that interacted with viral proteins. The comparative analysis of the H5N1 and H1N1 interacting proteins revealed a relatively high similarity of the host protein sets obtained by affinity tagging/purification (8–10). However, the similarity was reduced when comparing the affinity tagging/purification results with host interactors obtained by RNAi screening (21–23). Ultimately, it was evident that the host interactors of different subtypes of avian influenza virus were conserved to some extent, which indicated that different subtypes of AIV might use the same cellular machinery for replication.

In this study, we demonstrated that many host proteins interact with viral proteins in the form of complexes (Figure 2B), suggesting that the functions of these proteins are similar. For example, protein synthesis, transport-related proteins, and proteins involved in viral translation were closely linked, forming protein complexes that interacted with multiple viral proteins, and the host factors in these complexes included RPL26L1, RPL27, RPL18A, RPL21, RRBP1, RPS24, RPS3A, RPS2, RPS15A, GNB2L1, RPS20, and EIF3A. Other complexes included proteins involved in DNA replication and DNA repair, such as MCM3, MCM4, MCM5, MCM6, SMC2, SMC3, SMC4, SMC5, RFC2, LIG1, and NCAPG2 (Figure 2B), and proteins regulating ER to Golgi vesicle-mediated transport, such as COPA, COPG, ARCN1, ARF1, ARF4, and ARF6. In prior literature, eukaryotic translation initiation factor 3A (eIF3A) was found to positively affect the replication of vesicular stomatitis virus (26). Our results revealed that it participated in virus replication as a member of the interactive complex. The MCM complex, a cellular DNA replication licensing factor, is required for the replication of the influenza virus genome (27). Our results confirm MCM4–7 as interacting factors of the H5N1 avian influenza virus that may participate in viral replication. In addition, the SMC5/6 complex, a restriction factor that selectively blocks extrachromosomal DNA transcription, is associated with the hepatitis B virus genome and extrachromosomal reporters (28). Here, we identified SMC2–5 as proteins that interact with the H5N1 avian influenza virus. Finally, our results showed that the MCM and SMC complexes were also closely related in the global landscape of the virus and host interaction, suggesting that these two complexes may play a synergistic role in virus replication and transcription.

The pathway enrichment analysis among the host interactors of the H5N1 avian influenza virus revealed that the proteasome pathway was significantly enriched and was represented by USP5, USP10, and USP14. One important future direction of this work is to detail how these ubiquitin proteases affect influenza virus replication. The ubiquitin–proteasome system (UPS) interacts extensively with the avian influenza virus and is closely involved in the adaptation of the virus to its hosts (29). Several UPS proteins, including USP5, USP7, USP9, USP13, USP15, and USP22, are upregulated by Epstein–Barr virus infection or mitogen activation in normal T and B lymphocytes (30), suggesting the possible roles for these proteins in virus replication. Furthermore, USP14 is a negative regulator in antiviral responses because it directly deubiquitinates K63-linked ubiquitin on retinoic acid-inducible gene I (RIG-I), which is a critical RNA virus sensor that initiates the antiviral immune response (31).

Within a host cell, ribonucleoproteins are transported to the nucleus where transcription and replication occur, and mRNAs are transported to the cytoplasm and translated into protein (32). In this study, we confirmed that STAU2 was a host interactor of the H5N1 avian influenza virus and was capable of promoting virus replication by promoting the transport of *NS1* mRNA from the nucleus to the cytoplasm. NS1 is a late-expressing viral protein that has recently been extensively studied due to its multifunctionality, as it is involved in host innate immune defense, host and viral mRNA expression, apoptosis, viral RNA splicing, and morphogenesis. Most of these functions are dependent on the protein's interaction with host factors (33–35). In this study, 76 of the total 186 NS1 interacting proteins, including STAU2, were found to be specific interaction factors. That is, these 76 host proteins only interacted with the NS1 viral protein. Interaction is usually a prerequisite for related proteins to perform their functions. The interaction between NS1 and STAU2 may provide a possible physical means for STAU2 to promote the transport of *NS1* mRNA.

STAU2 belongs to the double-stranded RNA (dsRNA)-binding protein family and is involved in the transport and localization of mRNAs to various subcellular compartments and organelles (36, 37). St Johnston et al. originally identified the *Drosophila* RNA-binding protein Staufen (Stau) as an mRNA transport factor that is required for establishing the anterior–posterior axis of the embryo (38, 39). More recently, human Staufen was shown to promote HIV-1 replication through a specific interaction with HIV-1 pr55Gag and genomic RNA (40). In addition, it also interacts with HIV-1 Rev, which promotes viral replication by positively regulating its RNA export activity (41). In particular, Rev influences the subcellular localization of human STAU2. When STAU2 was overexpressed alone in HEK293T cells, it remains localized to the cytoplasm. In contrast, when STAU2 is co-expressed with Rev, a fraction of STAU2 localizes to the nucleoli along with Rev, indicating that the localization of STAU2 is influenced by the presence of Rev. The authors did not report further on how this interaction promotes the RNA export activity of Rev and, thus, viral production, but speculated that, together with CRM1 and STAU2, Rev may form an elaborate export complex for the successful transportation or regulated release of large viral RNAs across the nuclear membrane (40). STAU2 is involved in forming ribonucleoprotein complexes (RNPs) (41, 42) and in the targeted packaging and localization of mRNA in polarized cells (36). In mice, STAU1 and 2 are paralogs that share about 50% protein-sequence identity, and both are involved in mRNA localization (42, 43). In addition, the downregulation of *STAU2* in HCT116 colorectal cancer cells increases DNA damage and promotes apoptosis (44). STAU1 also specifically binds to the 5′-untranslated region of Enterovirus 71 viral RNA, promotes viral RNA replication (45), and promotes the replication of infectious bursal disease virus (IBDV) by binding to viral genomic double-stranded RNA and attenuating the MDA5-dependent induction of β interferon (46). These findings also demonstrate that although STAU2 or its homolog STAU1 is a double-stranded RNA-binding protein, it might also bind with the secondary structure of the single-stranded RNA formed by partially complementary sequences to then affect the replication of single-stranded RNA viruses, such as HIV virus and Ebola virus (41, 47). In the context of influenza, STAU1 was previously identified as an interacting factor of NS1 (48) and virus ribonucleoproteins, and it is required for the efficient replication of IAV (49). In this study, we uncovered that STAU2 interacted with the H5N1 avian influenza virus NS1 protein. We checked several host–viral interactome studies and found mass spectrometry results that identified STAU2 as a host interactor of the H1N1 influenza virus (9). Thus, we hypothesize that the interaction between NS1 and STAU2 might be a conserved association in different influenza strains. We further demonstrated that STAU2 promoted the export of *NS1* mRNA (Figure 5) from the nucleus to the cytoplasm in host cells of different species, which further promoted the replication of the influenza virus. Taken together, these findings underscore the important role of STAU2 as an interaction factor in virus replication.

## Data Availability Statement

The raw data supporting the conclusions of this article will be made available by the authors, without undue reservation.

## Author Contributions

QL and GZhao designed and supervised the study. QW, QL, QZhan, MZ, and JW analyzed the data. QW, QL, and QZhan performed the experiments. QW, QL, and GZhao wrote the paper.

## Conflict of Interest

The authors declare that the research was conducted in the absence of any commercial or financial relationships that could be construed as a potential conflict of interest.

## Supplementary Material

Supplementary Figure 1 Expression of all the viral genes in chicken cells. Whole cell lysate (input) was used as the positive control, IP indicates the immunoprecipitated enriched virus protein, and the IgG lane is the negative control, indicating that the antibody used for the IP was ordinary IgG with no specificity.

Supplementary Figure 2 Associations between H5N1 and H1N1 overlapping interacting proteins. The network nodes represent proteins. The edges represent protein-protein associations.

Supplementary Figure 3 *STAU2* knockdown did not affect the nuclear export of H5N1 AIV viral mRNAs other than *NS1*. 293T cells were infected with H5N1 AIV for 12 h, followed by separation into their nuclear and cytoplasmic fractions. The distribution of the viral mRNA between those fractions was detected by RT-PCR.

Supplementary Table 1 List of the host proteins that co-immunoprecipitated with 11 H5N1 AIV viral proteins.

Supplementary Table 2 Interactions between H5N1 AIV and the host proteins in chicken DF1 cells.

Supplementary Table 3 STRING interactions among the virus-interacting chicken proteins.

Supplementary Table 4 Venn diagram comparing the host interacting proteins of H5N1 AIV and H1N1 influenza virus.

Supplementary Table 5 Gene Ontology terms enriched among the H1N1 and H5N1 overlapping interacting proteins.

Supplementary Table 6 Pathway enrichments of H1N1 and H5N1 overlapping interacting proteins.
