# Supplementary figures and images for: Viral–Host Interactome Analysis Reveals Chicken STAU2 Interacts With Non-structural Protein 1 and Promotes the Replication of H5N1 Avian Influenza Virus
Source: Front Immunol. 2021 Apr 21;12:590679. doi: 10.3389/fimmu.2021.590679 (PMC8098808; doi:10.3389/fimmu.2021.590679)

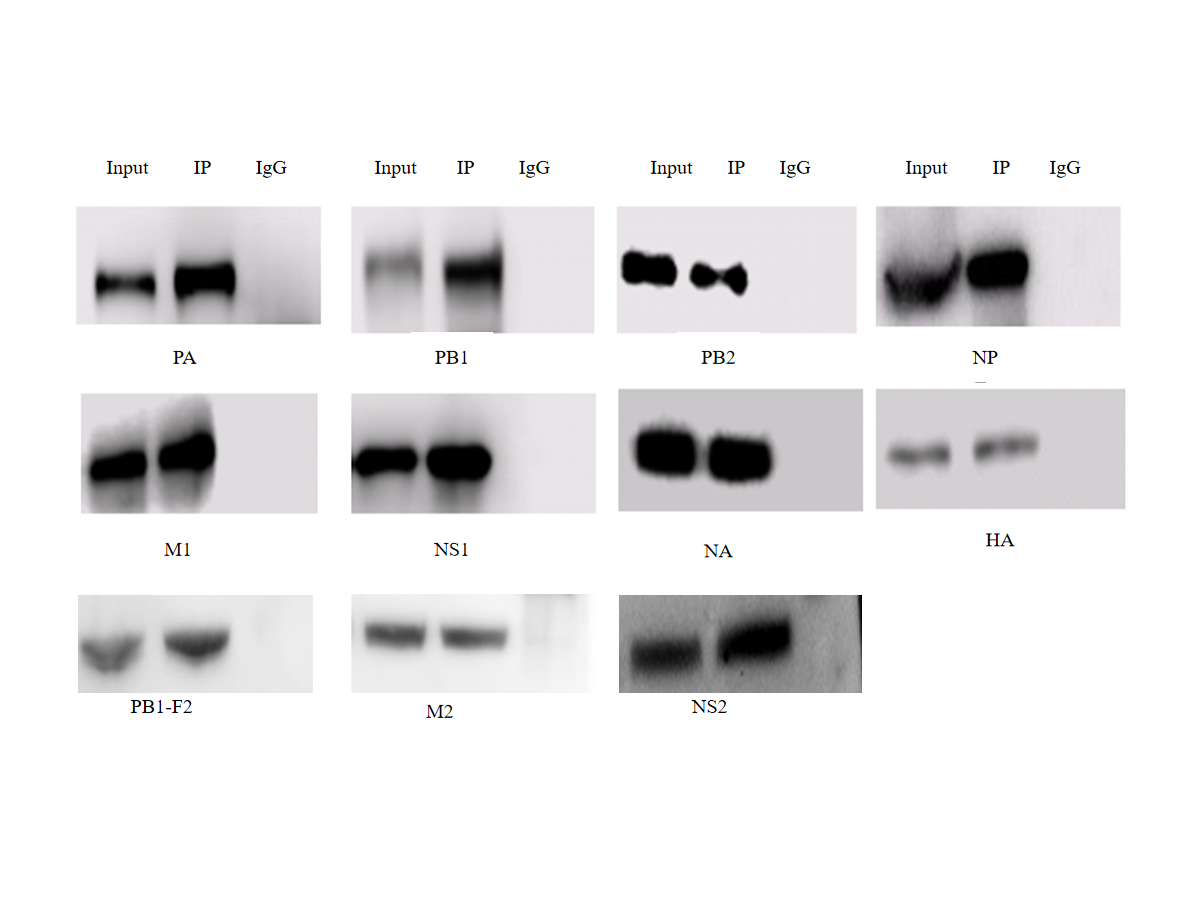

Supplement: Supplementary Figure 1 — Expression of all the viral genes in chicken cells. Whole cell lysate (input) was used as the positive control, IP indicates the immunoprecipitated enriched virus protein, and the IgG lane is the negative control, indicating that the antibody used for the IP was ordinary IgG with no specificity. [file Image_1.TIF]

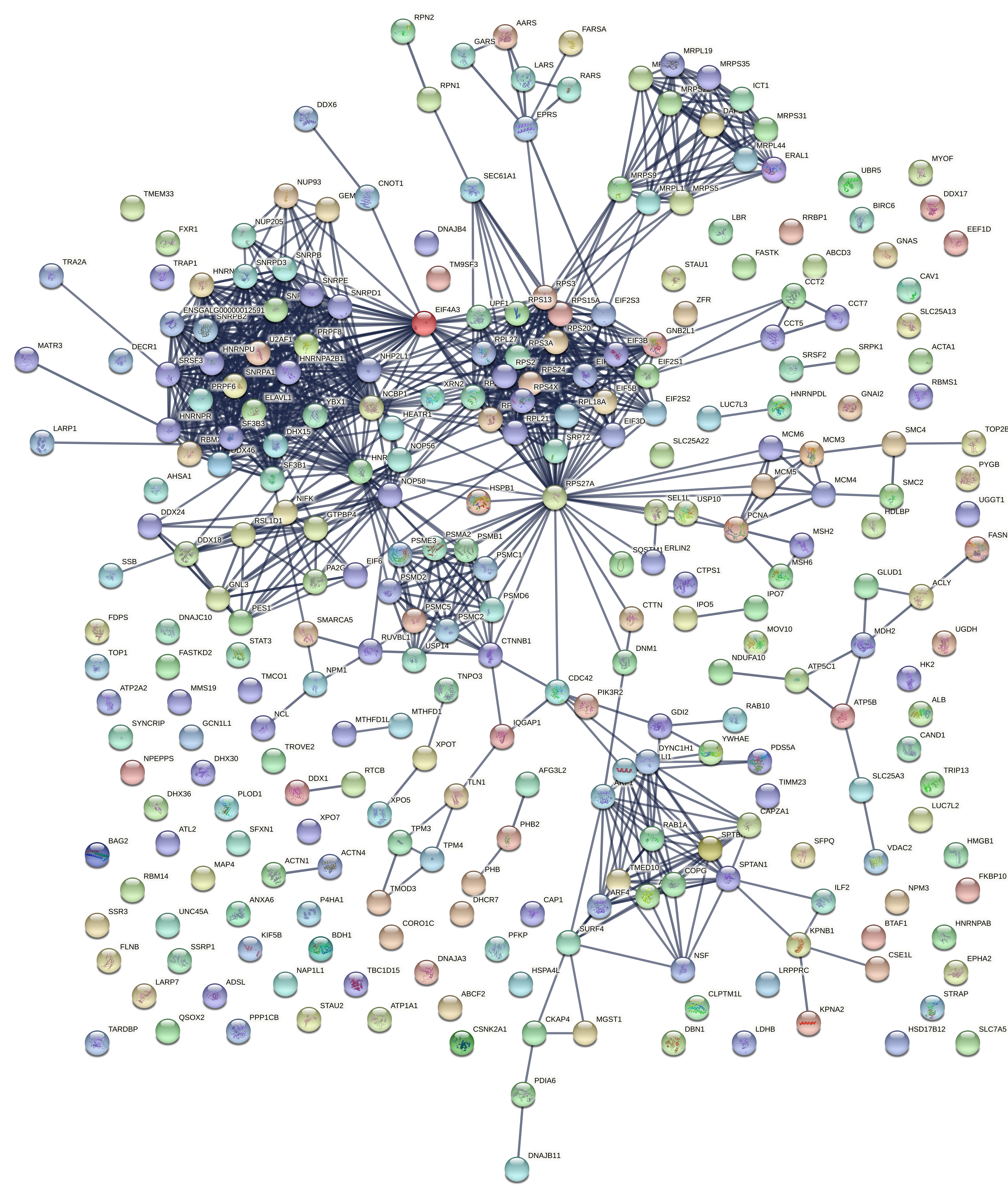

Supplement: Supplementary Figure 2 — Associations between H5N1 and H1N1 overlapping interacting proteins. The network nodes represent proteins. The edges represent protein-protein associations. [file Image_2.PNG]

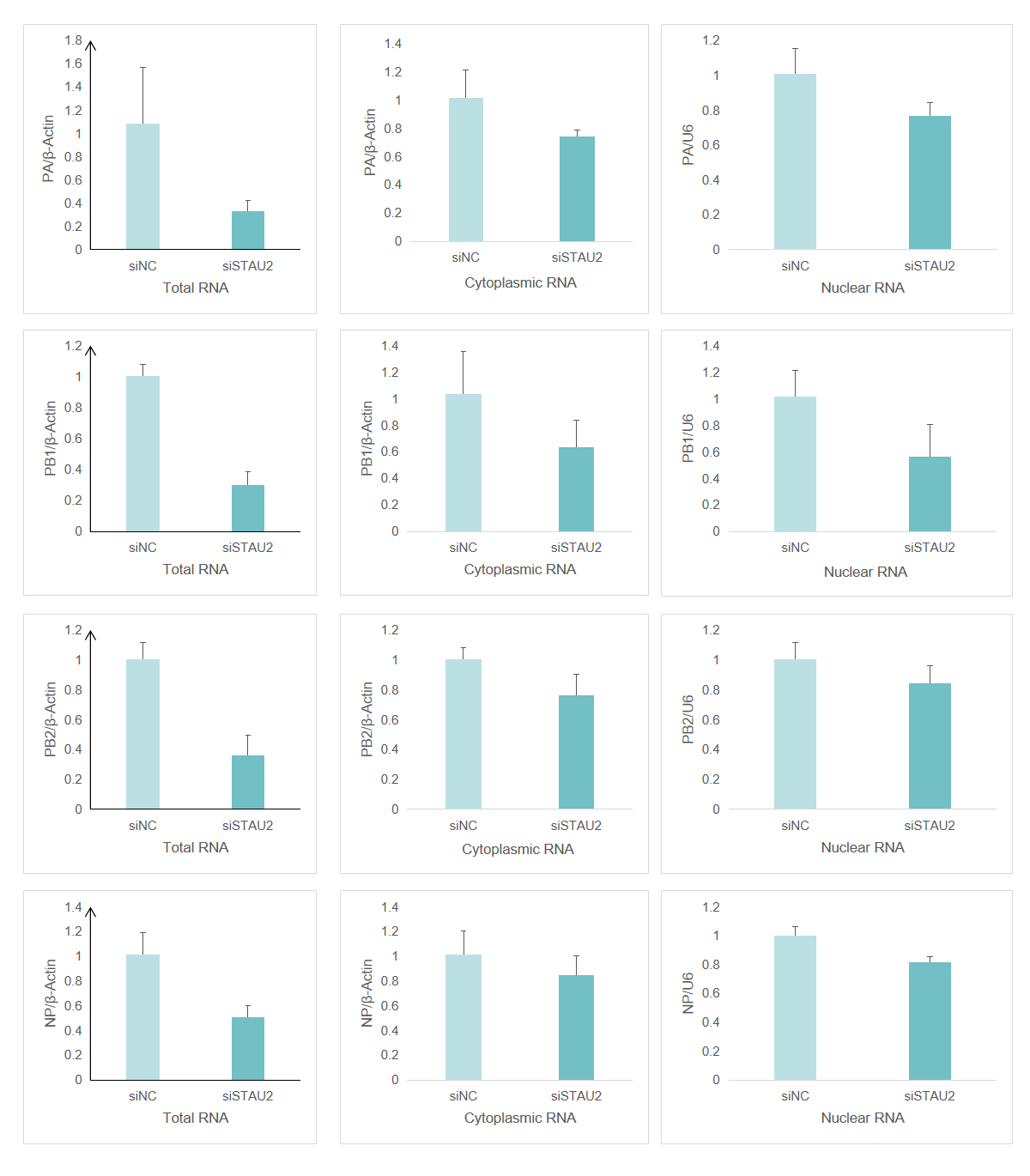

Supplement: Supplementary Figure 3 — STAU2 knockdown did not affect the nuclear export of H5N1 AIV viral mRNAs other than NS1. 293T cells were infected with H5N1 AIV for 12 h, followed by separation into their nuclear and cytoplasmic fractions. The distribution of the viral mRNA between those fractions was detected by RT-PCR. [file Image_3.TIF]
