# Supplementary material for: Viral–Host Interactome Analysis Reveals Chicken STAU2 Interacts With Non-structural Protein 1 and Promotes the Replication of H5N1 Avian Influenza Virus
Source: Front Immunol. 2021 Apr 21;12:590679. doi: 10.3389/fimmu.2021.590679 (PMC8098808; doi:10.3389/fimmu.2021.590679)
Supplement: Supplementary Table 5 — Gene Ontology terms enriched among the H1N1 and H5N1 overlapping interacting proteins. [file Table_5.DOC]

Table S5. Gene Ontology terms enriched among the H1N1 and H5N1 overlapping interacting proteins

| GO term | Count | Involved genes/Total genes (%) | *P* value |
| --- | --- | --- | --- |
| *Molecular function* |  |  |  |
| poly(A) RNA binding | 84 | 30.2 | 1.4E-37 |
| RNA binding | 27 | 9.7 | 1.5E-11 |
| ATP binding | 60 | 21.6 | 5.4E-10 |
| nucleotide binding | 23 | 8.3 | 0.000000024 |
| ATP-dependent RNA helicase activity | 11 | 4 | 0.000000089 |
| structural constituent of ribosome | 18 | 6.5 | 0.0000002 |
| mRNA binding | 11 | 4 | 0.000012 |
| double-stranded RNA binding | 8 | 2.9 | 0.000039 |
| translation initiation factor activity | 7 | 2.5 | 0.00035 |
| U2 snRNA binding | 3 | 1.1 | 0.0022 |
| oxidized purine DNA binding | 3 | 1.1 | 0.0044 |
| proteasome-activating ATPase activity | 3 | 1.1 | 0.0072 |
| GTPase activity | 9 | 3.2 | 0.014 |
| TBP-class protein binding | 3 | 1.1 | 0.015 |
| nuclear export signal receptor activity | 3 | 1.1 | 0.015 |
| *Biological process* |  |  |  |
| translation | 18 | 6.5 | 0.000000031 |
| osteoblast differentiation | 11 | 4 | 0.0000017 |
| RNA secondary structure unwinding | 8 | 2.9 | 0.0000047 |
| mRNA splicing, via spliceosome | 9 | 3.2 | 0.000059 |
| spliceosomal snRNP assembly | 6 | 2.2 | 0.00008 |
| IRES-dependent viral translational initiation | 4 | 1.4 | 0.00018 |
| positive regulation of RNA polymerase II transcriptional preinitiation complex assembly | 4 | 1.4 | 0.00034 |
| DNA repair | 10 | 3.6 | 0.00065 |
| negative regulation of translation | 6 | 2.2 | 0.00074 |
| protein folding | 9 | 3.2 | 0.001 |
| positive regulation of translation | 5 | 1.8 | 0.0011 |
| oxidation-reduction process | 10 | 3.6 | 0.0013 |
| protein import into nucleus | 6 | 2.2 | 0.0016 |
| positive regulation of telomerase RNA localization to Cajal body | 4 | 1.4 | 0.0026 |
| viral translational termination-reinitiation | 3 | 1.1 | 0.004 |
| DNA replication initiation | 4 | 1.4 | 0.0066 |
| response to virus | 5 | 1.8 | 0.0076 |
| regulation of translational initiation | 4 | 1.4 | 0.0079 |
| RNA processing | 5 | 1.8 | 0.0096 |
| positive regulation of protein localization to Cajal body | 3 | 1.1 | 0.0097 |
| DNA unwinding involved in DNA replication | 3 | 1.1 | 0.0097 |
| mRNA processing | 6 | 2.2 | 0.01 |
| *Cellular component* |  |  |  |
| membrane | 67 | 24.1 | 1.4E-25 |
| extracellular exosome | 84 | 30.2 | 2.9E-15 |
| nucleoplasm | 61 | 21.9 | 5.6E-13 |
| nucleolus | 28 | 10.1 | 0.00000086 |
| intracellular ribonucleoprotein complex | 9 | 3.2 | 0.0000011 |
| nuclear chromosome, telomeric region | 9 | 3.2 | 0.00026 |
| MCM complex | 4 | 1.4 | 0.00045 |
| proteasome regulatory particle, base subcomplex | 4 | 1.4 | 0.0015 |
| cytoplasmic ribonucleoprotein granule | 4 | 1.4 | 0.002 |
| nuclear pore | 5 | 1.8 | 0.0028 |
| cytoplasmic mRNA processing body | 6 | 2.2 | 0.0029 |
