# Supplementary material for: Viral–Host Interactome Analysis Reveals Chicken STAU2 Interacts With Non-structural Protein 1 and Promotes the Replication of H5N1 Avian Influenza Virus
Source: Front Immunol. 2021 Apr 21;12:590679. doi: 10.3389/fimmu.2021.590679 (PMC8098808; doi:10.3389/fimmu.2021.590679)
Supplement: Supplementary Table 6 — Pathway enrichments of H1N1 and H5N1 overlapping interacting proteins. [file Table_6.DOC]

Table S6. Pathway enrichments of H1N1 and H5N1 overlapping interacting proteins

| **Term** | **Count** | **Involved genes/Total genes (%)** | **P value** |
| --- | --- | --- | --- |
| Spliceosome | 23 | 8.3 | 7.9E-12 |
| Ribosome | 18 | 6.5 | 0.00000075 |
| RNA transport | 17 | 6.1 | 0.000015 |
| Proteasome | 8 | 2.9 | 0.00024 |
| Protein processing in endoplasmic reticulum | 14 | 5 | 0.0021 |
| Ribosome biogenesis in eukaryotes | 8 | 2.9 | 0.0091 |
| Aminoacyl-tRNA biosynthesis | 6 | 2.2 | 0.017 |
| DNA replication | 5 | 1.8 | 0.022 |
| Adherens junction | 6 | 2.2 | 0.097 |
